# mRNA-miRNA networks identify metabolic pathways associated to the anti-tumorigenic effect of thyroid hormone on preneoplastic nodules and hepatocellular carcinoma

**DOI:** 10.3389/fonc.2022.941552

**Published:** 2022-09-20

**Authors:** Marina Serra, Rajesh Pal, Elisabetta Puliga, Pia Sulas, Lavinia Cabras, Roberto Cusano, Silvia Giordano, Andrea Perra, Amedeo Columbano, Marta Anna Kowalik

**Affiliations:** ^1^ Department of Biomedical Sciences, Unit of Oncology and Molecular Pathology, University of Cagliari, Cagliari, Italy; ^2^ Department of Oncology, University of Turin, Turin, Italy; ^3^ Candiolo Cancer Institute-Fondazione del Piemonte per l'Oncologia (FPO), Istituto di Ricovero e Cura a Carattere Scientifico (IRCCS), Candiolo, Italy; ^4^ Centro di Ricerca, Sviluppo e Studi Superiori in Sardegna (CRS4), Pula, Italy

**Keywords:** Hepatocarcinogenesis, Thyroid hormone, Keap1-Nrf2, OXPHOS, miR-182

## Abstract

**Background:**

Thyroid hormones (THs) inhibit hepatocellular carcinoma (HCC) through different mechanisms. However, whether microRNAs play a role in the antitumorigenic effect of THs remains unknown.

**Methods:**

By next generation sequencing (NGS) we performed a comprehensive comparative miRNomic and transcriptomic analysis of rat hepatic preneoplastic lesions exposed or not to a short-term treatment with triiodothyronine (T3). The expression of the most deregulated miRs was also investigated in rat HCCs, and in human hepatoma cell lines, treated or not with T3.

**Results:**

Among miRs down-regulated in preneoplastic nodules following T3, co-expression networks revealed those targeting thyroid hormone receptor-β (Thrβ) and deiodinase1, and Oxidative Phosphorylation. On the other hand, miRs targeting members of the Nrf2 Oxidative Pathway, Glycolysis, Pentose Phosphate Pathway and Proline biosynthesis – all involved in the metabolic reprogramming displayed by preneoplastic lesions– were up-regulated. Notably, while the expression of most miRs deregulated in preneoplastic lesions was not altered in HCC or in hepatoma cells, miR-182, a miR known to target *Dio1* and mitochondrial complexes, was down-deregulated by T3 treatment at all stages of hepatocarcinogenesis and in hepatocarcinoma cell lines. In support to the possible critical role of miR-182 in hepatocarcinogenesis, exogenous expression of this miR significantly impaired the inhibitory effect of T3 on the clonogenic growth capacity of human HCC cells.

**Conclusions:**

This work identified several miRNAs, so far never associated to T3. In addition, the precise definition of the miRNA-mRNA networks elicited by T3 treatment gained in this study may provide a better understanding of the key regulatory events underlying the inhibitory effect of T3 on HCC development. In this context, T3-induced down-regulation of miR-182 appears as a promising tool.

## Introduction

The thyroid hormones (THs), thyroxine (T4) and 3,3′,5-triiodo-L-thyronine (T3) influence a variety of physiological processes, including development, metabolism, cell growth and proliferation (1). Although it has been proposed that rapid non-genomic mechanisms initiated at the cell membrane could mediate some actions of thyroid hormones (2), most of the effects of THs on cellular proliferation and differentiation are driven by the thyroid hormone nuclear receptors (THRs) THRα and THRβ (3, 4).

Liver, where THRβ represents the most abundant isoform (5, 6), is an important target organ of THs and growing evidence implicates THs and THRs in HCC development. Indeed, three independent case-control studies suggested that hypothyroidism represents a risk factor for human HCC (7–9). As to non-alcoholic steatohepatitis (NASH), a well-known pro-tumorigenic condition, several works showed that subclinical and clinical hypothyroidism and reduced THRβ expression correlated with more progressed stages (10–13), although this correlation has been questioned by other studies that found a positive association of free T3 levels with the severity of hepatic steatosis and fibrosis (14, 15). Experimental and clinical studies revealed a status of severe local hypothyroidism in rat hepatic preneoplastic lesions, and in rat and human HCCs (16–18), suggesting that this condition may represent a favorable event for HCC development. Accordingly, T3 exogenous administration not only inhibited liver tumor formation but also induced regression of HCCs *in vivo* (19). The effect of T3 has been attributed to several mechanisms, including its ability to induce mitophagy, differentiation and metabolic reprogramming of pre- and neoplastic cells (19–21). In particular, it was shown that T3 can induce a switch of preneoplastic hepatocyte gene expression profile towards that of fully differentiated cells (19). Among the mechanisms responsible for regulating gene expression are microRNAs (miRs), single-stranded and highly conserved non-coding RNAs which can negatively control the expression of several target genes, resulting in the regulation of at least 30% of protein-coding genes (22). MiRs also play a role in cancer pathogenesis, acting either as oncogenes or tumour suppressor genes (23, 24).

In this context, increased levels of miR-21, -146a, -181a and -221, all predicted to target THRβ, were found in papillary thyroid cancer (PTC) patients, in association with low levels of THR*β* transcripts (25); moreover, *THRβ* expression in human clear cell renal carcinomas (ccRCC) was inversely correlated with that of miR-204 (26). Nevertheless, although a number of studies showed that miRNAs may be involved in repressing *THR*β expression (25–28), which are the miRs targeted by TRs and what is their role in normal and neoplastic hepatocytes remain elusive. Since accumulating evidence demonstrate that aberrant expression of miRNAs represents a very frequent event in human HCC (29, 30), by Next Generation Sequencing (NGS) we performed a comprehensive and comparative analysis of the expression of mRNAs and miRNAs in preneoplastic rat livers undergoing regression following treatment with T3. We found that T3 induced deregulation of several miRs controlling the expression of genes associated to pathways involved in metabolic reprogramming, such as Oxidative Phosphorylation (OXPHOS), Nrf2-mediated stress response, glycolysis, pentose-phosphate pathway (PPP) and proline biosynthesis. Among these miRs, miR-182 was the only miR deregulated by T3 at all the stages of hepatocarcinogenesis and in HCC cell lines. Interestingly, exogenous expression of miR-182 partially hampered the inhibitory effect of T3 on the clonogenic ability of HCC cells.

## Material and methods

### Resistant-hepatocyte model

Guidelines for Care and Use of Laboratory Animals were followed during the investigation. All animal procedures were approved by the Ethical Commission of the University of Cagliari and the Italian Ministry of Health (N. 1247/2015-PR 16/10/2015). Male Fischer F-344 rats (100-125g) were purchased from Charles River (Milano, Italy). As reported in **Supplementary Figure 1**, animals were subjected to the Resistant-Hepatocyte (R-H) model of hepatocarcinogenesis, consisting of a single intraperitoneal dose of diethylnitrosamine (150 mg/kg body weight, DEN, Sigma), followed by a brief (2 weeks) promoting procedure with 2-acetylaminofluorene (2-AAF, Sigma) and a two-thirds partial hepatectomy (PH) (31). Rats were then switched to a basal diet for 5 weeks. After 9 weeks from DEN, rats were split into two groups: one group was fed a T3-supplemented diet (4 mg/kg of diet) for 4 days, while the second group of animals was maintained on a basal diet. Rats kept on a basal diet for 10 weeks were used as a control group. Another group of animals exposed to the R-H protocol was maintained on basal diet for 10 months, a time when all rats developed HCC. Animals were then split into two groups; one group was fed T3 supplemented diet for 1 week while the other was kept on basal diet. Histologic classification of preneoplastic nodules and HCCs was performed as previously described (19, 32).

### Immunohistochemistry

Frozen liver sections were collected, cut into 6µm and fixed in formalin for 6 hours at room temperature and stained for hematoxylin eosin (H&E) and the placental form of glutathione S-transferase (GSTP). Paraffin-embedded sections were incubated overnight with the following antibody: anti-GSTP (MBL, Nagoya, Japan), anti-NQO1 (Abcam, ab28947), anti-KRT-19 (NB100-687, Novus Biologicals) and anti‐rabbit or anti-mouse Dako EnVision+^®^ System Labelled Polymer‐HRP (Dako Corporation, Carpinteria, CA). Peroxidase binding sites were detected by VECTOR^®^ NovaRED™ Peroxidase (HRP) Substrate Kit (Vector Laboratories).

### Laser capture micro-dissection

Sixteen-μm-thick serial frozen sections of rat livers were attached to 2-μm RNase free PEN-membrane slides (Leica, Wetzlar, Germany). Microdissection (Leica, LMD6000) was preceded by a H&E and GSTP staining on serial sections.

### RNA and miRNA isolation

Total RNA was isolated with the mirVana miRNA isolation kit (Life Technologies) from 3 livers of untreated rats, and preneoplastic nodules from rats treated with T3 (6 nodules) or not exposed to the hormone (5 nodules). As to HCC, total RNA was isolated from 11 HCCs from rats subjected to a 1-week T3 feeding or 10 HCCs from rats not exposed to the hormone. RNA was quantified by Nanodrop spectrophotometer (Thermo Scientific) and its integrity was evaluated by Agilent Bioanalyzer 2100. Only RNA samples with a RIN (RNA Integrity Number) ≥ 7 were included in the study.

### Deep sequencing and data processing

For RNA and miRNA sequencing experiments, indexed libraries were prepared using 100 ng of total RNA as starting material, with a TruSeq Stranded Total RNA Sample Prep Kit and QIAseq miRNA Library Kit (Illumina Inc.) respectively. Libraries were sequenced (single-end, 75 cycles) at a concentration of 8 pM/lane on the HiSeq 3000 platform (Illumina Inc.). Raw RNA-seq reads were quality assessed and trimmed for sequencing adaptor using TrimGalore (v0.6.5) with the default Phred score. Pre- alignment quality control was performed using FASTQC. Reads were subsequently mapped to UCSC rat genome build (rn6) using STAR (v2.7.3a). The mapped reads were then indexed using samtools (v1.10) to extract the overlapping alignments. Reads aligning to the transcripts were counted and quantified using kallisto (V0.46.2). Additionally, post- alignment quality control and gene-body coverage analysis was performed using RseQC. Raw miRNA reads were preprocessed using FASTQC for quality control. Further, reads with Unique Molecular Identifiers (UMI) and low quality base calls were trimmed off using UMI-tools and TrimGalore (v0 6.5) respectively. Processed reads were mapped to the reference rat genome build (rn6) downloaded from UCSC using Bowtie. The R/Bioconductor package “DESeq2” was used to identify differentially expressed genes and miRNAs. Genes and miRNAs with adjusted P-value of 0.05 were considered for further analysis.

### qRT-PCR

Gene expression was assessed in HCCs by qRT-PCR using specific Taqman probes (*Nqo1*,Rn00566528_m1; *Gstp1*, Rn00561378_gH; *Dio1*, Rn00572183_m1; *Krt-19*, Rn0149686*7_*m1*).* Real-time PCR for PYCR1 and ALDH18A1 was performed in HepG2 cells using SYBR Green (SsoAdvanced Universal SYBR^®^ Green Supermix). Gene-specifc primer sequences were as follows: PYCR1: forward (5’- CATCTGCTCATTCACGCACT), reverse (5’- AACCTATGTGGGGAGCACAG), ALDH18A1: forward (5’-TGTGGAGGGGAAGAAAGTTG) and reverse (5’- CAGATCAGCCAGATGATGGA). Each sample was run in triplicate and gene expression analysis of Glyceraldehyde 3-phosphatase dehydrogenase (*Gapdh*) or β*-actin* were used as reference genes. Analysis of miRNA-expression: cDNA was synthesized using the TaqMan^®^ MicroRNA Reverse Transcription Kit. qRT-PCR amplification was performed with the reverse transcription product, TaqMan^®^ 2X Universal PCR Master Mix, No AmpErase ^®^UNG. MiR primers used were: hsa-miR-185, 002271; hsa-miR-425-5p, 001516; hsa-miR-27a, 000408; rno-miR-224, 464298, mmu-miR-182, 002599. Probe mix was from Thermo Fisher Scientific. The endogenous control U6 (U6 snRNA, 001973) was used to normalize miRNA expression levels.

### Cell cultures and *in vitro* experiments

HepG2 cell line was obtained from ATCC (Manassas, VA, USA). Mahlavu cells were a kind gift of Dr. N. Atabey; the mutational status of this cell line can be found at the site https://lccl.zucmanlab.com/hcc/cellLines/Mahlavu. Cells were routinely cultured in essential aminoacids supplemented-MEM and DMEM medium (Sigma-Aldrich; Saint Louis, MO, USA), respectively, in the presence of 10% fetal bovine serum, P/S (100U/ml Penicillin, 100mg/l Streptamicin), and L-Glutamine (2mM) (Lonza, Basel, Switzerland) and incubated at 37°C in a 5% CO2-95% air-humidified atmosphere. Cells were transduced with either a lentiviral vector expressing THRB gene (GeneCopoeia, cat. EX-T9450-Lv186) or an empty lentiviral vector. Both cell lines were seeded in the presence or in the absence of 100 nM triiodothyronine (Sigma) for 48 hours. Analysis of miRNA expression was performed as described (17). Briefly, the expression of miR-140 (hsa-miR-140-3p, #002234), miR-185 (hsa-miR-185-5p, #002271), miR-425 (hsa-miR-425-5p, #001516), miR-421 (hsa-miR-421, #002700), miR-224 (hsa-miR-224-5p, # 483106_mir), and miR-182 (hsa-miR-182, # 002334) in Mahlavu and HepG2 cells, was performed starting from equal amounts of total RNA/sample (50ng) using the specific Taqman microRNA assay or TaqMan Advanced miRNA Assays kits (Applied Biosystems). MiRNA expression was calculated as fold change using the delta-delta CT method and RNU48 as endogenous control. miR-191-5p, consistently expressed in HepG2 cells was used to normalize mir-224-5p levels (33).

### miR-182 infection

For miR-182 stable expression, 7×10^4^ Mahlavu THRB cells were seeded in six well plates and transduced with miR-182 or control pGIPZ lentiviral vectors (kindly provided by Dr. Wei, Northwestern University School of Medicine, Chicago, US (34). Anchorage-independent growth, for Mahlavu THRB cells was performed by seeding 3000 cells/well in 0.5% soft agar containing 10% charcoal T3-stripped serum supplemented DMEM. T3 was added to the medium at a concentration of 100nM three times per week for 15 days. Grown colonies were visualized by iodonitrotetrazolium chloride staining (Sigma) and counted using ImageJ software.

### Ingenuity pathway analysis

For identifying genes associated with the canonical pathways, disease and functions and gene networks, Ingenuity Pathway Analysis (IPA^®^, Redwood City-CA) was used. Core analysis was performed on the dataset with Ingenuity knowledge base and species selected to mouse and rat only. For identifying the target genes of differentially expressed miRNA microRNA Target filter module was used. Two distinct statistical analyses were performed during the core analysis. A right tailed fisher’s exact test was used to determine the probability that each biological function enriched in the dataset is statistically significant and not due to the chance alone (p < 0.05). Additionally, Z-score was calculated to provide predictions about upstream or downstream biological process. In the present study, -log(p-value) of 1.3 (corresponding to 0.05) and z score of ≥ 2 and ≤ -2 was considered for further analysis.

### Statistics

Data are expressed as mean ± standard deviation (SD). Analysis of significance was done by Student’s *t-*test using the GraphPad software (La Jolla, California).

## Results

**Figure 1:**
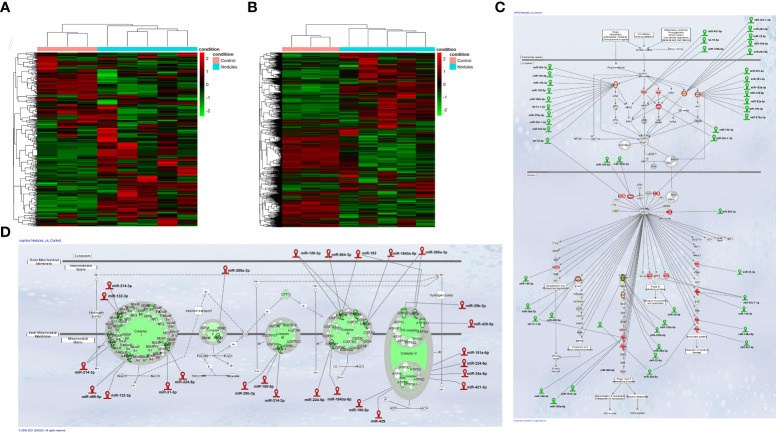
**(A)** Hierarchical clustering of miRNAs in preneoplastic lesions (nodules), and control samples. Each row represents the expression of a gene and each column a sample. Red and green colors represent higher or lower mRNA expression levels (median-centered), respectively; **(B)** Hierarchical clustering of genes in preneoplastic lesions (nodules), and control samples. Each row represents the expression of a gene and each column a sample. Red and green colors represent higher or lower mRNA expression levels (median-centered), respectively; **(C)** IPA analysis of the Nrf2 oxidative stress response pathway and; **(D)** the Oxidative Phosphorylation pathway (OXPHOS) in early preneoplastic nodules. Hairpin loop structures represent miRNAs. (Red: Up-regulated, Green: Down-regulated). Edges connect miRNAs and putative target genes predicted by TargetScan/TarBase/miRecords and Ingenuity Expert Findings.

Integrative miRNA and mRNA analysis stratifies preneoplastic lesions and control liver into two clusters with different expression profile. Study design was organized to allow the comparison of both small non-coding RNA and gene expression profiles in the same preneoplastic nodules from rats subjected to a single initiating dose of DEN followed by a promoting procedure (R-H model) (31) (**Supplementary Figure 1A**). In our initial analysis, we performed mRNA and miRNA NGS expression profiling in preneoplastic nodules *vs*. control liver. Unsupervised hierarchical cluster analysis of miRs stratified control livers and preneoplastic nodules into two clearly defined groups (**Figure 1A**). Accordingly, principal components analysis (PCA) separated the samples into two groups (**Supplementary Figure2A**). Analysis of differentially expressed miRNAs evidenced that 88 miRNAs (cut off ≥ 1.4) were modified in their expression, with 49 of them being up-regulated (**Supplementary Table 1**). Notably, 13 among the 15 most down-regulated miRNAs have already been described as oncosuppressors in experimental or human HCCs (35–42), whereas some of the differentially up-regulated miRNAs were similarly regulated in human HCC (miR-181, miR-183-5p, miR-421 and miR-21) (43–46); finally, several miRNAs of uncertain function were never described in relation with liver cancer. MiR-224-5p, the most up-regulated miR in nodules (+254 fold *vs*. controls), was recently shown to target deiodinase 1 (Dio1), an enzyme expressed in the liver and kidney and required for the conversion of T4 to T3 in rat hepatocytes and human renal carcinoma cells (47, 48). Moreover, at least other two up-regulated miRs - miR-421-3p and miR-185-5p - are also predicted (Target Scan), but not experimentally validated, to target Dio1, further supporting the notion that local hypothyroidism can favour progression to HCC (17). Gene expression profiling was performed in the same samples. A total of 4599 differentially expressed genes (fold-change ≥ 1.5) were identified in nodules *vs*. control livers: 2372 genes were up-regulated and 2227 were down-regulated (**Supplementary Table 2**). Similar to what observed for miRNAs, unsupervised hierarchical cluster analysis stratified control livers and preneoplastic nodules into two separate groups (**Figure 1B**) and PCA separated the samples into two groups (**Supplementary Figure 2B**). Ingenuity pathway analysis (IPA) revealed that the top 5 most dysregulated pathways were LPS/IL-1-mediated inhibition of RXR function, FXR/RXR activation, Mitochondrial dysfunction, Nrf2-mediated oxidative stress response and OXPHOS (**Supplementary Figure 2C**). To identify a possible correlation between miRNAs and genes differentially expressed in preneoplastic nodules *vs*. control livers, we investigated miRNAs potentially targeting genes dysregulated in our transcriptomic analysis. The results showed that a high percentage of modified genes are targets of dysregulated miRNAs. Of particular interest was the finding that several miRNAs targeting genes involved in the most altered pathways (i.e. Nrf2 pathway and OXPHOS) were inversely regulated compared to their target genes. Indeed, 29 miRs predicted to target genes involved in the strongly activated Keap1-Nrf2 pathway were down-regulated in preneoplastic nodules; conversely, 18 miRs up-regulated in the nodules were reported to target genes involved in OXPHOS, a pathway profoundly down-regulated in preneoplastic lesions (**Figures 1C, D**).

### T3 treatment profoundly affects gene expression profile of preneoplastic nodules

**Figure 2:**
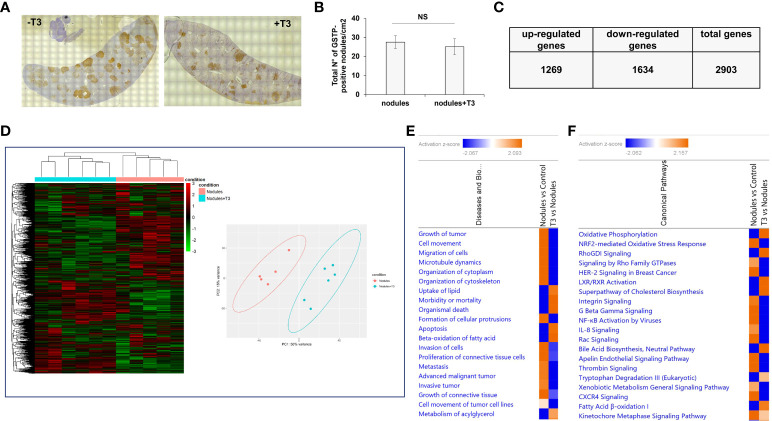
*T3 modifies the global gene expression profile of preneoplastic lesions.* **(A)** An overview of GSTP immunohistochemical staining of representative preneoplastic lesions of untreated (-T3) or 4-day T3 treated animals (+T3); **(B)** Number of GSTP-positive preneoplastic nodules in rat livers subjected to the R-H protocol and then treated with T3 or not for 4 days; **(C)** Up- and down-regulated genes in nodules of rats fed T3 for 4 days *vs*. preneoplastic nodules of untreated animals; **(D)** Hierarchical clustering of genes in untreated preneoplastic lesions (nodules) or treated with T3 for 4 days (nodules+T3). Each row represents the expression of a gene and each column a sample. Red and green colors represent higher or lower mRNA expression levels (median-centered), respectively (Left), and Principal component analysis (PCA) indicative of the variability of gene expression data in nodules of rats untreated or treated with T3 for 4 days (right); **(E)** Top 20 diseases and functions identified by IPA core analysis in untreated nodules *vs*. control and T3-treated nodules *vs.* untreated nodules; **(F)** Top 20 Canonical Pathways identified by IPA Core Analysis in untreated nodules *vs*. control and T3-treated nodules *vs.* untreated nodules. Color is determined by Z-score; the Z-score >2 and <-2 is considered meaningful. Blue color indicates suppressed disease/biological function or canonical pathways; orange indicates activated disease/biological function or canonical pathways.

As previously described (49), treatment with T3 for 7 days induced a rapid regression of preneoplastic nodules (ca. 70%). To investigate the molecular changes causing this regression, we performed an NGS analysis of laser-microdissected preneoplastic nodules 4 days after T3 treatment, a time that precedes the disappearance of the nodules, and when the number of preneoplastic lesions is still similar to that of untreated rats (**Figures 2A, B** **)**. To identify the potential transcriptional changes induced by T3 treatment, we performed transcriptomic analysis. In T3-treated nodules 2903 genes were differentially expressed (fold-change ≥ 1.5), 1269 being up-regulated and 1634 down-regulated (**Figure 2C** and **Supplementary Table 3**). Unsupervised hierarchical cluster analysis stratified again untreated nodules and T3-treated nodules into two clearly distinct clusters and PCA separated the samples according to the two groups (**Figure 2D**). Disease and biological function analysis of the differentially expressed (DE) genes revealed remarkable enrichment in several functional categories that were reversed upon T3 treatment (**Figure 2E**). Top 5 categories down-regulated upon T3 treatment were Growth of tumor, Cell movement, Migration of cells, Microtubule dynamics and Organization of cytoplasm. Differentially expressed genes were also categorized based on Canonical Pathways. The two most significantly dysregulated pathways upon T3 treatment were OXPHOS and Nrf2-mediated Oxidative Stress Response (**Figure 2F**), two pathways dysregulated, but in the opposite direction, in nodules *vs*. control livers (**Figures 1C, D** **)**.

### Integrative analysis of miRNA-mRNA expression profiles unveils T3-induced modification of metabolic pathways

**Figure 3:**
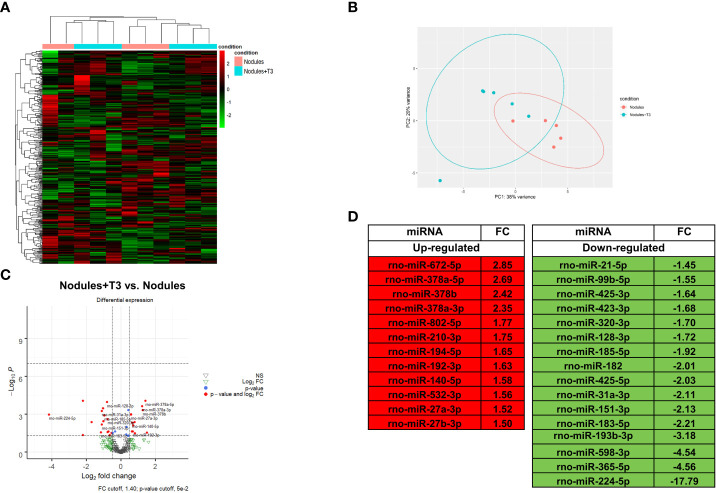
*T3 modifies the global miRNA expression profile of preneoplastic lesions* **(A)** Hierarchical clustering of miRNA in preneoplastic lesions untreated (nodules) or treated with T3 for 4 days (nodules +T3). Each row represents the expression of a gene and each column a sample. Red and green colors represent higher or lower miRNA expression levels (median-centered), respectively; **(B)** Principal component analysis (PCA) indicative of the variability of miRNA expression data in nodules untreated or treated with T3 for 4 days; **(C)** Volcano plot of the 28 significantly differentially expressed miRNAs. Red dots indicate the miRNAs which qualify the P-value of 0.05 and Fold Change (FC) 1.4. Green and grey triangles indicate miRNAs which did not pass the P-value and Fold change cutoff respectively; **(D)** List of miRNAs differentially up- and down-regulated in nodules of rats fed T3 for 4 days *vs*. nodules of untreated animals.

MiR NGS analysis was performed in the same nodules analyzed for transcriptomics. Unsupervised hierarchical cluster and PCA analyses stratified the samples into two different clusters, with the exception of two samples (**Figures 3A, B** **)**. Twenty eight miRNAs (cut off ≥ 1.4) were differentially expressed, 12 of them being up-regulated (**Figures 3C, D** **)**. MiRs have been reported to act by binding to the promoter region, and to specific sequences in UTR and coding regions. Interaction with the promoter region has been reported to preferentially promote transcription, while binding to the UTR sites to predominantly induce transcriptional repression (22–24). In this study, we analysed only miRNAs binding to the UTR sites. A state of local hypothyroidism represents a promoting condition for the progression of preneoplastic lesions to HCC (17), and restoration of a normal T3/THR axis is associated to their regression (19, 49). However, whether miRs are involved in the increased expression of THRβ-target genes is unclear. Therefore, we also investigated whether relevant changes in miRNAs targeting Thrβ, Dio1 or other genes involved in the activation of the THR/RXR pathway take place after T3. Our analysis showed down-regulation of miR-224-5p and miR-185-5p, two miRs known (47, 48) or predicted (TargetScan) to target Dio1. Notably, miR-224-5p - which was the most up-regulated in the nodules when compared to controls (254-fold) - was also the most-down-regulated following T3 treatment; conversely, transcriptomic analysis performed in the same nodules (**Supplementary Table 3**) showed that Dio1 was up-regulated in T3-treated (12-fold increase) compared to untreated nodules. Dio1 up-regulation was also associated with a concomitant decreased expression of miR-182 (-2.01-fold change) and miR-185-5p (-1.92-fold change). According to the NGS analysis, qRT-PCR validation showed T3-induced down-regulation of all the three examined miRs (185-5p, 182 and 224-5p (**Supplementary Figure 3**). The possible role of miRs in the reversion from a local hypo- to a hyperthyroid state of the nodules was also supported by the finding that miR-425-5p - predicted to target THRβ and up-regulated in preneoplastic nodules - was down-regulated by T3 treatment (**Figure 3D**) in parallel with enhanced THRβ mRNA levels (**Supplementary Table 3**). No inverse correlation between miRs and THRβ expression was observed for other miRs previously suggested as THRβ regulators, such as miR-155 (50).

### T3 up-regulates miRs targeting genes involved in the Nrf2-mediated oxidative stress response

**Figure 4:**
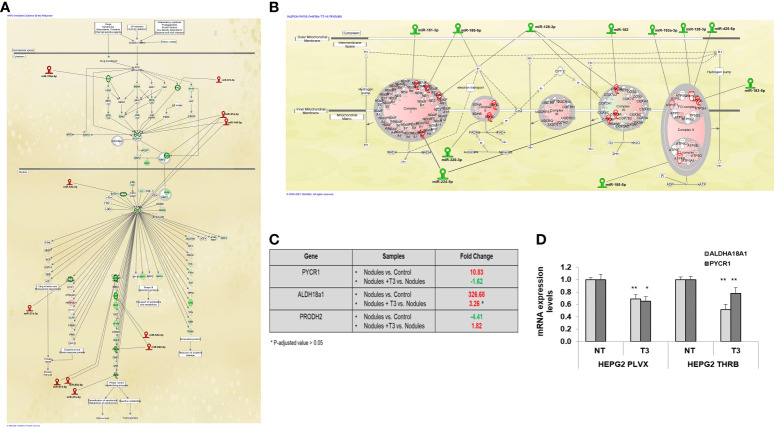
*MiRNA:mRNA integrated network and proline biosynthesis in preneoplastic nodules following T3 treatment* **(A)** IPA analysis of Nrf2 oxidative stress response pathway in T3 treated nodules *vs*. untreated nodules; **(B)** IPA analysis of Oxidative Phosphorylation pathway in T3-treated nodules *vs*. untreated nodules; **(C)** Results of NGS analysis showing fold change of the key genes involved in proline metabolism: *Pycr1, Aldh18a1 and Prodh2. P*-adjusted value = 0.05 and FC ± 1.5; **(D)** qRT-PCR analysis of the expression of *PYCR1 and ALDH18a1* in HepG2 cells not transduced (PLVX) or transduced with THRB and treated (T3) or not (NT) for 48 hours with 100nM T3. MiRNA expression was calculated as fold change using the 2^-ΔΔCt^ method and RNU48 as endogenous control. Student t-test: *P < 0.05; **P<0.01. Values are expressed as mean ± SD.

Next, we investigated the involvement of miRs in the deregulation of the pathways most modified by T3 treatment, and in particular the Nrf2 and the OXPHOS pathways. Among the miRNAs whose expression was up-regulated by T3 treatment, we found a number of miRs known or predicted to target genes involved in the activation of the Nrf2 pathway, previously shown to promote HCC development in humans as well as in animal experimental models (51). Up-regulation of Nrf2 has been observed in the experimental model herein used and T3 treatment was shown to reverse Nrf2 activation, although the mechanisms underlying this effect were not defined (32). The present NGS analysis revealed that most Nrf2-target genes were down-regulated by T3 treatment (**Figure 4A**), and, interestingly, this effect was associated with up-regulation of 6 miRs predicted (miR-27a-3p, 378a-5p, 532-3p, 802-5p, 672-5p) or shown (miR-140-5p) to target Nrf2 or genes involved in the Keap1-Nrf2 pathway (52). It is also worth to mention that the decreased expression of glucose 6-phosphate dehydrogenase (G6pd), another Nrf2 target implicated in the metabolic reprogramming of preneoplastic lesions, was paralleled with up-regulation of miRNA-672-5p (**Figure 4A**).

### T3 down-regulates miRs targeting genes involved in OXPHOS

Another pathway strongly modified by T3 treatment was the OXPHOS pathway that was strongly down-regulated in preneoplastic nodules (for a comparison see **Figures 4B** and **1D**). Among the miRs whose expression was down-regulated by T3 treatment, we found 9 miRs predicted to target the five mitochondrial complexes (miR-151-3p, 185-5p, 128-3p,182, 193a-3p, 128-3p, 425-5p, 320-3p, 224-5p). Notably, the induction of miR-182 has also been reported to promote glucose metabolism in non-small lung cancer cells (53). Thus, the T3-induced switch from glycolysis to OXPHOS may be mediated, at least in part, by down-regulation of these miRs.

### T3 down-regulates proline biosynthesis pathway

Together with the switch from glycolysis to OXPHOS, T3 treatment also led to inhibition of proline biosynthesis, recently shown to be required for HCC progression (54). Indeed, as demonstrated in **Supplementary Tables 2**, **3**, and **Figure 4C**, the expression of *Pycr1* and *Aldh18a1*, two enzymes involved in proline synthesis, was strongly up-regulated in preneoplastic nodules and down-regulated following treatment with T3. To directly establish whether T3 could affect proline biosynthesis, we analyzed the expression of *PYCR1* and *ALDH18a1* in THRβ-transfected HepG2 cells. As shown in **Figure 4D**, T3 significantly diminished the expression of both the genes in mock as well as in THRβ-transfected cells. Notably, among the miRs induced by thyroid hormone was miR-672-5p, predicted to target *Pycr1* (**Figure 3D**).

### T3 down regulates the expression of miR-182 in rat HCCs and human hepatocarcinoma cell lines

**Figure 5:**
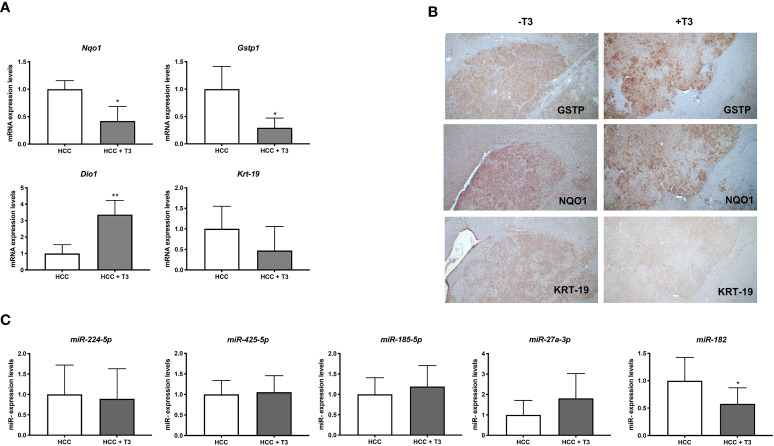
QRT-PCR analysis of miRs and mRNAs involved in T3/THR axis and Nrf2 pathway following T3 treatment in HCC. **(A)** qRT-PCR analysis of the expression of *Gstp, Nqo1, Dio1 and Krt-19* in HCC bearing rats. Rats exposed to the R-H protocol were sacrificed 10 months after treatment with a single dose of DEN. The week prior to sacrifice, one group of animals was fed a T3-supplemented diet as described in Materials and Methods. Each sample was run in triplicate and gene expression analysis of beta-actin was used as endogenous control. Relative quantification analysis for each gene was calculated by 2^-ΔΔCt^ method. Student t-test: *P<0.05; **P<0.01; Values are expressed as mean ± SD; **(B)** GSTP, NQO1 and KRT-19 immunohistochemistry of HCCs from livers of animals treated as described in B (X5); **(C)** qRT-PCR analysis of the expression of miR-224, miR-425-5p, miR-185, miR-182, and miR-27 in HCC bearing rats. Each sample was run in triplicate and U6 snRNA was used as endogenous control. Relative quantification analysis for each gene was calculated by 2^-ΔΔCt^ method. Student t-test: *P<0.05. Values are expressed as mean ± SD.

**Figure 6:**
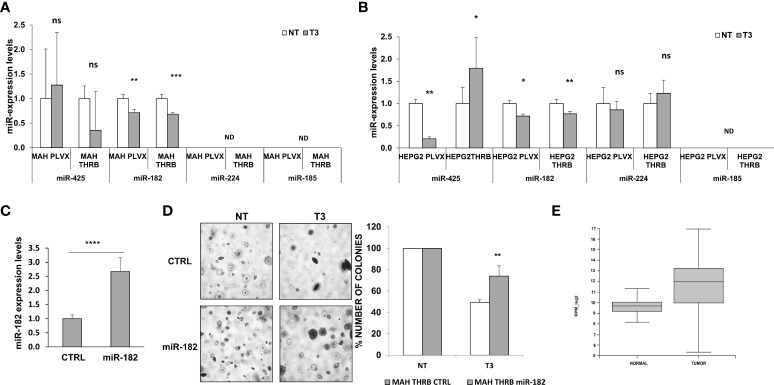
*Expression of miRs in HCC cell lines and effect of miR-182 transfection on anchorage- independent growth of HCC cells.* **(A)** Expression of miR-140, 185, 425 and 421, 224 and 182 in Mahlavu or **(B)** HepG2 cells not transduced (PLVX) or transduced with THRB and treated (T3) or not (NT) for 48 hours with 100nM T3. Each sample was run in triplicate and RNU48 was used as endogenous control. Relative quantification analysis for each gene was calculated by 2^-ΔΔCt^ method; ND: not detected. Values are expressed as mean ± SD; **(C)** qRT-PCR analysis of miR-182 following transfection of Mahlavu THRB cells. RNU48 was used as endogenous control. Relative quantification analysis for miR-182 was calculated by 2^-ΔΔCt^ method. Student t-test: ***P<0.001. Values are expressed as mean ± SD; **(D)** Anchorage‐independent growth of Mahlavu THRB cells described in **(A)** Visible colonies were counted after 15 days (Left), and Graph representing the number of colonies of Mahlavu THRB cells described in A stably transduced with miR-182 or a control (shSCR) and grown in anchorage-independent conditions (Right). Student t-test: *P < 0.05; **P < 0.01; ***P < 0.001. Values are expressed as mean ± SD; **(E)** Relative expression of mir-182-5p in normal and tumor group in TCGA (the blue represents normal group, the orange represents tumor group). The expression level is represented in RPM_log2 values. Mann-Whitney U-test: ****P < 0.0001. The expanded form of "ns" is: not significant.

Preneoplastic nodules are considered, as a population, the precursors of HCC. However, it is known that not all the genetic/epigenetic changes found at early stages of hepatocarcinogenesis are maintained until the final steps of this process (55, 56). For this reason, we investigated whether the expression of miRs associated to T3-induced metabolic reprogramming of preneoplastic nodules is affected by T3 also at late stages of hepatocarcinogenesis. To this aim, we analyzed by qRT-PCR the expression of some of the genes/miRNAs engaged in the T3/THR axis or in the Nrf2 pathway in laser-microdissected HCCs generated 10 months after DEN (**Supplementary Figure 1B**) and characterized by their positivity to cytokeratin-19 (*Krt-19*) (**Figures 5A, B**), a marker of the most aggressive rat and human HCCs (57, 58). The results showed that while the expression of genes implicated in the Nrf2 pathway (*Nqo1, Gstp1)*, T3/THR axis (*Dio1*) was affected by T3 in a similar manner in preneoplastic nodules and HCCs (**Figures 5A, B** and **Supplementary Tables 2**, **3**), only the expression of miR-182 but not that of the other examined miRs (miR-224-5p; miR-425-5p; miR-185-5p; miR-27a-3p), was modified in HCCs following treatment with T3 (**Figure 5C** and **Supplementary Table 2**). These results suggest that the effect of T3 on Nrf2 pathway, OXPHOS or in the T3/THR axis at late stages of hepatocarcinogenesis is no longer dependent upon the vast majority of these miRNAs. To further explore whether miRs could play a role in the T3-modified molecular pathways in HCC, we analyzed the expression of miRNAs involved in the T3/THR axis and in the Nrf2 pathway in two different human HCC cell lines, HepG2 and Mahlavu transduced with THRβ, with and without T3 treatment. As shown in **Figure 6A**, differently from what was observed in T3-treated preneoplastic nodules, but similar to HCC, no change of the expression of miRNAs involved in Nrf2 pathway, OXPHOS and T3/THR axis (miR-140, miR-185, miR-421, miR-425 and miR-224) was detected in mock or THRβ−transfected HepG2 or Mahlavu cells exposed to thyroid hormone, again with the only exception being miR-182. Similar results were found in Mahlavu-THRβ cells (**Figure 6B**).

### MiR-182 overexpression impairs the anti-tumorigenic effect of T3

Since the results showed that miR-182 was the only miR significantly deregulated by T3 at both early and late stages of hepatocarcinogenesis, as well as in the HCC cell lines, we investigated its involvement in the growth of HCC cells. To establish whether the anti-tumorigenic role of T3 could be due, at least in part, to its inhibitory effect on miR-182 expression, we transiently transduced the cells with miR-182 (**Figure 6C**), and then determined their anchorage-independent growth with and without T3 treatment. As shown in **Figures 6C, D**, while T3 strongly inhibited the growth of untransduced tumor cells, exogenous miR-182 expression severely affected the T3-inhibitory effect. To further support the translational value of our *in vivo* and *in vitro* data we performed a database analysis of miR-182 expression in normal liver tissue (50 samples) and liver hepatocellular carcinoma (LIHC, 372 samples). The TCGA data analysis demonstrated a significant (P-value 2.2e -16) up-regulation of miR-182 in LIHC (median of 827.16 rpm in normal *vs* 4017.64 rpm in tumor group) (**Figure 6E** **).**

## Discussion

MiRNAs are a large family of endogenous, small-non coding RNAs that regulate gene expression at post transcriptional level. In the last years, they have increasingly gained importance due to their widespread occurrence and diverse functions as regulatory molecules in all aspects of cancer biology, such as proliferation, invasion, apoptosis, and angiogenesis (22–24). Importantly, miRNAs appear to play a critical role not only in established HCC but also at the initial steps of hepatocarcinogenesis, as revealed in both experimental and human studies (30). Previous works reported that the anti-tumorigenic effect of T3 on rat hepatic preneoplastic nodules and HCC was associated with the ability of T3 treatment to revert the Warburg phenotype and induce a differentiation program of pre- and neoplastic hepatocytes (49). However, the mechanism(s) through which T3 reprograms poorly differentiated hepatocytes to a more mature phenotype leading to the disappearance of preneoplastic nodules is not fully understood. To answer this question, we carried out in-depth analysis of miRNAs and mRNAs in preneoplastic nodules following T3 treatment. The present work unveiled miRNA-mRNA pairs implicated in the control of pathways involved in the regression of preneoplastic nodules. We identified 28 miRNAs differentially expressed in T3 treated preneoplastic lesions compared to those not exposed to thyroid hormone and the most significantly deregulated pathways controlled by these miRs. Among the major deregulated pathways, we identified OXPHOS, and Nrf2-mediated oxidative stress response. OXPHOS down-regulation and activation of glycolysis are well-established hallmarks of proliferating liver cancer cells, as they dramatically reprogram some of the metabolic pathways to meet the increased energetic and anabolic needs (59). In this context, T3 treatment for 4 days led to down-regulation of 8 miRs predicted/shown to target genes of membrane mitochondrial complexes; down-regulation of these miRs was associated to up-regulation of 90% of the genes involved in the mitochondrial respiratory chain and to restoration of OXPHOS. Among the down-regulated miRNAs, 4 have been reported to act as Onco-miRNAs in HCC; indeed, miR-224, miR-425, miR-151-3p, miR-182-5p and miR-320-3p have been shown to promote cell proliferation, invasion, and metastasis (60–63). Interestingly, miR-224, predicted to target the Ubiquinone Oxidoreductase Subunit A8 and the Cytochrome c oxidase subunit 6B1, has been reported as an early-stage biomarker in HCC patients (64) and as an accelerator of HCC progression (65). We also observed that MiR-185-5p and miR-320-3p, predicted to target Succinate Dehydrogenase Complex Subunit C (SDHC) and D (SDHD), respectively, were down-regulated in T3 treated nodules, further supporting the recovery of OXPHOS observed following T3 treatment. Accordingly, we observed up-regulation of the Succinate dehydrogenase complex, a tetramer consisting of SDHA, SDHB, SDHC and SDHD subunits that exerts a critical tumor suppressive role and has been proposed as an important therapeutic target in HCC (66). Additionally, up-regulation of mir-320-3p has also been implicated in early stages of steatohepatitis, a condition favoring HCC development (67). Cancer cell proliferation leads to the accumulation of *Reactive Oxygen Species* (*ROS*) which activate the Keap1-Nrf2 signaling pathway. The Keap1-Nrf2 system has been reported to protect cancer cells from DNA-damage (68). In preneoplastic nodules, T3 treatment significantly up-regulated 6 miRNAs predicted/shown to target genes involved in this pathway. Interestingly, 3 of these miRs (378a-3p, 27a-3p and 140-5p) are known to play a tumor suppressive role during HCC progression. Indeed, miR 378a-3p has been reported to be a tumor suppressor in human HCC (69) probably by causing G2/M cell cycle arrest (70), whereas miR 27a-3p and 140-5p – are predicted to target Nrf2 - inhibit cell viability and migration in HCC (71, 72).

The present study also confirms that T3 is able to revert the local hypothyroid status of preneoplastic and neoplastic hepatocytes and suggests that some miRs (i.e. miR-224 and miR-185-5p) may play a role in restoring the euthyroid status, at least at early stages of tumorigenesis. Studies on metabolic alterations in cancer have mainly focused on aerobic glycolysis and central carbon metabolism, including the citric acid cycle and the pentose phosphate pathway. Nevertheless, several reports suggested that amino acids may also play a relevant role to support survival and proliferation of cancer cells (73). In this context, recent studies demonstrated that modulation of the expression of the enzymes involved in proline biosynthesis significantly influences proliferation of HCC cells *in vitro* and tumor formation *in vivo* (54). Our analysis enabled us to show that T3, in addition to a switch from glycolysis to OXPHOS, profoundly modified proline biosynthesis by inhibiting - on the one hand - the expression of two enzyme critically involved in proline synthesis and - on the other hand - up-regulating *Prodh* which promotes proline catabolism. Of note, these effects were observed in preneoplastic nodules and in HCCs and as well as in HepG2-THRβ transfected cells. Altogether, these results provide novel information on the anti-tumorigenic role of T3, suggesting thyroid hormone as a critical player in interfering with metabolic pathways altered in HCC development. Intercrossing miRs-mRNAs data revealed that T3-induced regression of preneoplastic nodules was accompanied by deregulation of a number of miRs targeting genes involved in metabolic pathways altered at early stages of HCC development; however, our data also point out that most of the miRs found dysregulated at early stages were not modified by T3 at the final steps of tumorigenesis. An interesting exception is represented by miR-182. This miR - predicted to target both *Dio1* as well as members of the mitochondrial complexes – was found down-regulated at all stages of hepatocarcinogenesis and in hepatocarcinoma cells upon T3 treatment. Interestingly, exogenous expression of miR-182 in HCC cells significantly hampered the inhibitory effect of T3 on clonal expansion of cancer cells, thus supporting the role of this miR in the carcinogenic process and providing an additional mechanism to the anti-tumorigenic effect of T3. It is puzzling that, in spite of the ability of T3 to induce similar biological effects (namely, regression) both in preneoplastic nodules and in established HCCs (19), as suggested by our data, the underlying molecular mechanisms are likely not overimposable. To gain further insights on this important aspect, similar miR/mRNA analyses in the context of HCC, performed in the very same lesions, are advisable. It is expected that the integration of all these data could lead to the identification of key pathways/molecules responsible for the ability of T3 to induce regression of pre/neoplastic hepatic lesions that can thus be proposed as new therapeutic targets.

## Data availability statement

The datasets presented in this study can be found in online repositories. The names of the repository/repositories and accession number(s) can be found in the article/**Supplementary Material**.

## Ethics statement

All animal procedures were approved by the Ethical Commission of the University of Cagliari and the Italian Ministry of Health (N. 1247/2015-PR/10/2015).

## Author contributions

Manuscript concept: AC, MK, AP. Drafting the manuscript: MS, PS, RP, MK. Experimental design: PS, RP, MK, MS, EP. Acquisition of research data: PS, RP, MK, EP, LC, RC, MS. Analysis of research data: MS, PS, AC, SG, RC, AP. Organization of research data: AC, AP, SG. Interpretation of the data: AC, AP, SG. Critical review and revision of the manuscript: AC, AP, MK, SG. All authors contributed to the article and approved the submitted version.

## Funding

This work has been supported by the Associazione Italiana Ricerca sul Cancro (AIRC, Grants IG-15279 to AC and IG-20210 to SG), and Regione Autonoma Sardegna (RAS to AC).

## Conflict of interest

The authors declare that the research was conducted in the absence of any commercial or financial relationships that could be construed as a potential conflict of interest.

## Publisher’s note

All claims expressed in this article are solely those of the authors and do not necessarily represent those of their affiliated organizations, or those of the publisher, the editors and the reviewers. Any product that may be evaluated in this article, or claim that may be made by its manufacturer, is not guaranteed or endorsed by the publisher.

## Supplementary material

Supplementary Figure 1 Experimental Protocols. **(A)** Rats were given a single dose of DEN (150 mg/kg bw) followed by a 2-weeks feeding of 2-acetylaminofluorene (0.02%) and a two-thirds partial hepatectomy (PH). Animals were then switched to a basal diet. Five weeks after 2-AAF withdrawal, one group of rats was fed a T3-supplemented diet (4 mg/kg of diet) for 4 days. Rats kept on a basal diet were used as a control group; **(B)** Rats given a single dose of DEN and then exposed to 2-AAF + PH as in A were switched to basal diet for 10 months. One group of animals was fed a T3-supplemented diet (4 mg/kg of diet) for 7 days prior to sacrifice. Rats kept on a basal diet were used as control group.

Supplementary Figure 2
**(A)** PCA of miRNAs in preneoplastic nodules and control samples**; (B)** PCA of genes in preneoplastic lesions (nodules), and preneoplastic nodules control samples; **(C)** Canonical pathway analysis in preneoplastic lesions (nodules), and control samples.

Supplementary Figure 3 qRT-PCR analysis of miR-185-5p miR-182 and miR-224 in GSTP- positive preneoplastic nodules in rat livers subjected to the R-H protocol and then treated with T3 or not for 4 days. U6 snRNA was used as endogenous control. Relative quantification analysis for miR-182 was calculated by 2^-ΔΔCt^ method. Student t-test: ***P* < 0.01. Values are expressed as mean ± SD.
